# Breast lesion detection using an anchor-free network from ultrasound images with segmentation-based enhancement

**DOI:** 10.1038/s41598-022-18747-y

**Published:** 2022-08-30

**Authors:** Yu Wang, Yudong Yao

**Affiliations:** 1 College of Medicine and Biological Information Engineering, Northeastern University, Shengyang, China; 2 Department of Electrical and Computer Engineering, Stevens Institute of Technology, Hoboken, USA

**Keywords:** Biomedical engineering, Cancer imaging, Cancer imaging

## Abstract

The survival rate of breast cancer patients is closely related to the pathological stage of cancer. The earlier the pathological stage, the higher the survival rate. Breast ultrasound is a commonly used breast cancer screening or diagnosis method, with simple operation, no ionizing radiation, and real-time imaging. However, ultrasound also has the disadvantages of high noise, strong artifacts, low contrast between tissue structures, which affect the effective screening of breast cancer. Therefore, we propose a deep learning based breast ultrasound detection system to assist doctors in the diagnosis of breast cancer. The system implements the automatic localization of breast cancer lesions and the diagnosis of benign and malignant lesions. The method consists of two steps: 1. Contrast enhancement of breast ultrasound images using segmentation-based enhancement methods. 2. An anchor-free network was used to detect and classify breast lesions. Our proposed method achieves a mean average precision (mAP) of 0.902 on the datasets used in our experiment. In detecting benign and malignant tumors, precision is 0.917 and 0.888, and recall is 0.980 and 0.963, respectively. Our proposed method outperforms other image enhancement methods and an anchor-based detection method. We propose a breast ultrasound image detection system for breast cancer detection. The system can locate and diagnose benign and malignant breast lesions. The test results on single dataset and mixed dataset show that the proposed method has good performance.

## Introduction

Breast cancer is one of the most prevalent type of cancer in women. According to the global cancer epidemic statistics released by the International Agency for Research on Cancer of the World Health Organization, there are approximately 2.89 million new female breast cancer cases worldwide each year, accounting for 24.2% of the total female cancer cases, ranking first^1^. Meanwhile, breast cancer incidence in developed countries is high, while the relative mortality in the less developed countries is the highest^2^. Clinical reports show that early detection and breast cancer treatment can significantly improve the survival rate^3^.

Mammography, digital breast tomosynthesis (DBT) and ultrasound imaging are three common imaging methods in clinical examination of breast cancer. However, mammography has the disadvantages of low specificity, high cost, and radioactivity^4^. Radioactivity causes health risks for patients, high cost increases the financial burden of patients, and low specificity (65–85%) leads to unnecessary biopsy operation^4^. DBT also has the disadvantages of high cost and radioactivity. In contrast, ultrasound imaging has the advantages of real-time imaging, no ionizing radiation, and low cost, and is commonly used in breast cancer screening or diagnosis. However, the diagnosis of ultrasound image is highly dependent on the skill level of the technician. Doctors with different training and different clinical experiences may make different diagnosis results^5^. Moreover, ultrasound images have high noise, significant artifacts, and low contrast between tissue structures. Therefore, it is desirable to develop a computer-aided breast cancer diagnosis system that can assist doctors in diagnosis.

**Figure 1:**
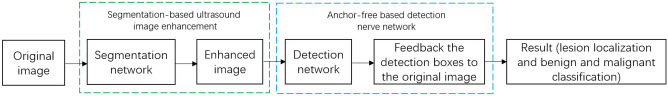
Our proposed breast lesion detection system.

Many researchers have studied the ultrasound diagnosis of breast cancer. Previous researches mainly applied traditional digital image processing techniques and machine learning technique to implement breast cancer detection^6,7^. For example, Drucker et al.^8^ first used radial gradient index filtering to detect the initial points of a region, examined the candidate areas from the background by maximizing the regional average radial gradient index of detection point growth, and classified the lesions using Bayesian neural networks. Finally, it achieved sensitivity of 87% at 0.76 false positive detection. As the most popular machine learning method, deep learning (DL) has gained a good reputation in computer vision and pattern recognition. In the medical field, many researchers have successfully applied DL to breast cancer detection^9–13^. Cao et al.^14^ comprehensively compared five object detection networks based on deep learning (Fast R-CNN^15^, Faster R-CNN^16^, you only look once (YOLO)^17^, YOLO V3^18^, and single shot multibox detector (SSD)^19^), and demonstrated that SSD achieved the best performance in terms of precision and recall. In a study on breast lesion detection, Yap et al.^20^ used Faster R-CNN as their deep learning network. To reduce the impact of small sample datasets on the experiment, they applied transfer learning. At the same time, they proposed a three-channel fusion method, the original image, the sharpened image, and the contrast enhanced image (three single-channel images), are merged into a new three-channel image. However, the limitations of the prior work include: (1) they did not explore the impact of image preprocessing on experimental results; (2) their datasets^14,20^ are not publicly available and other researchers can not conduct comparative experiments; and (3) they all used anchor-based object detection networks and they did not examine the impact of anchor size settings on the experimental results. Therefore, we address the above issues by proposing an anchor-free object detection method for breast cancer detection. In addition, a segmentation-based enhancement (SBE) method is proposed for the detection performance improvement. The system flow chart is shown in Fig. 1. We focus on improving the contrast of ultrasound images and improving the detection precision of breast lesions. The key contributions include:

1. We designed a segmentation-based ultrasound image contrast enhancement method.
2. We explore the use of an anchor-free object detection network to detect breast cancer, avoiding the complex calculations of the anchor-based detection network.
3. We propose a method of making object detection label by using lesion shape label.

The remainder of this paper is as follows, “Results” section presents the experimental results. “Discussion” and “Conclusions” section discuss and conclude our research, respectively. “Methods” section describes our experimental methods and procedures in detail.

## Results

We evaluated the performance of our breast lesion detection system using various datasets. We also compared with many different enhancement methods and detection networks. The performance metrics and experimental results are described bellow.

### Overview of datasets and breast lesion detection system

#### Datasets

**Figure 2:**
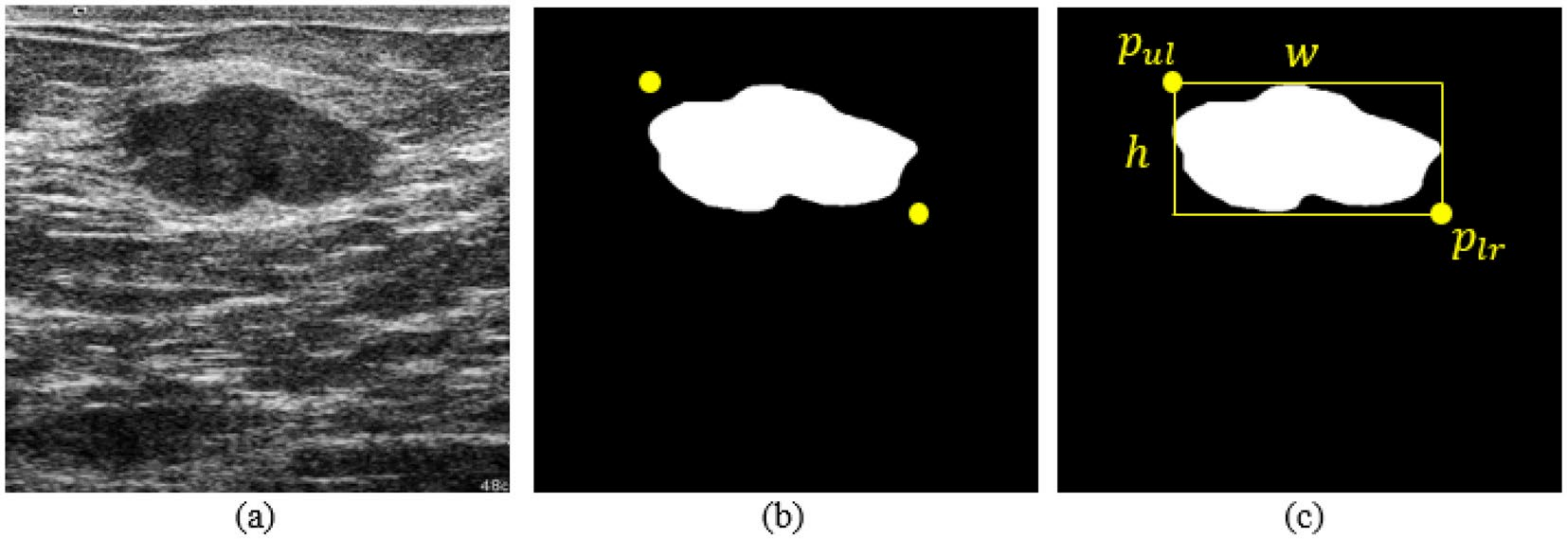
(**a**) Original ultrasound images; (**b**) ground truth in binary mask, yellow points represent the upper left and lower right corners of the ground truth; (**c**) represents a bounding box made according to the yellow points.

In this study, we used three public datasets, namely breast ultrasound (BUS)^21^, breast ultrasound image dataset (BUSI)^22^, and breast ultrasound image segmentation dataset (BUSIS)^23^. BUS was collected from the UDIAT Diagnostic Centre of the Parc Tauli Corporation, Sabadell (Spain). BUS contains 163 breast ultrasound images, of which 109 are benign and 54 are malignant. BUSI was collected from Baheya Hospital for Early Detection and Treatment of Women’s Cancer, Cairo, Egypt. The breast ultrasound images were collected from 600 female patients between 25 and 75 years old. BUSI contains 437 benign images, 210 malignant images, and 133 normal breast images, for a total of 730 breast ultrasound images. BUSIS was collected from the Second Affiliated Hospital of Harbin Medical University, the Affiliated Hospital of Qingdao University, and the Second Hospital of Hebei Medical University. BUSIS contains 562 images among women between 26 and 78 years old. These datasets contain multiple images for the same patient. The specific information of the datasets are shown in Table 1. In terms of image labels, BUS and BUSI include lesion shape labels and lesion benign and malignant classification labels (as shown in Fig. 2a,b), while BUSIS only contains lesion shape labels. In this study, we used BUSIS for image preprocessing and BUS and BUSI for breast lesion detecion.

#### Labels

The task of breast lesion detection is to identify and locate the exact localization of the lesion. Identification is to classify benign and malignant lesions and location is to give localization information of the lesion area. In BUS and BUSI datasets, the category labels of the lesions have been given, but there is no coordinate information of the lesions. We propose a method to obtain the lesion coordinates according to the lesion shape labels. As shown in Fig. 2b, we traverse all non-zero pixels in Fig. 2b, and find the largest and smallest horizontal and vertical coordinates $x_{\min }$, $x_{\max }$, $y_{\min }$, $y_{\max }$ among these non-zero pixels. We can obtain the upper left point $p_{ul} = (x_{\min }, y_{\min })$ and the lower right point $p_{lr} = (x_{\max },y_{\max })$ of the lesion area. The lesion area’s width *w* equal $x_{\max } - x_{\min }$ and height *h* equal $y_{\max } - y_{\min }$. We are then able to determine a bounding box of the lesion (Fig. 2). Finally, we use the five information set of $p_{ul}$, $p_{lr}$, *w*, *h* and lesion category as the label for breast lesion detection. However, in BUSIS dataset, because the lesion category is not given, it can not be used as the breast lesion detection data. Therefore, we use BUSIS in the image preprocessing step and we will introduce the use of BUSIS dataset in detail in the next section.

**Table 1:** A comparison of BUS, BUSI, and BUSIS.

Dataset	Total	Benign	Malignant	Normal	Label	Capture devices
BUS	163	109	54	–	Lesions shape and type	Siemens ACUSON Sequoia C512
BUSI	730	437	210	133	Lesions shape and type	LOGIQ E9 and LOGIQ E9 Agile
BUSIS	562	–	–	–	Lesions shape	GE VIVID 7, LOGIQ E9, Hitachi EUB-6500, Philips iU22, and Siemens ACUSON S2000

#### Overview of breast lesion detection system

Our system consists of two parts, the image preprocessing part and the breast lesion detection part. First, in the image preprocessing part, we use a new image enhancement method named segmentation-based enhancement (SBE). A deep learning method is used to segment the breast lesion region, and the segmented image is multiplied with the original image to obtain an enhanced image. Second, we input the enhanced image to an anchor-free object detection network (i.e., fully convolutional one-stage object detection network (FCOS)^24^) to detect the breast lesion.

### Performance metrics

We used Precision, Recall, and mean average precision (mAP) as the performance metrics in our experiments. The calculation of Precision, Recall, and mAP depends on the following parameters.

- IoU, in medical image analysis, IoU is also known as Jaccard Similarity Index or Jaccard Index. The IoU is defined by:
  1 $$\begin{aligned} \text {IoU}=\dfrac{{\text {Area of Overlap}}}{{\text {Area of Union}}}. \end{aligned}$$
  Among them, Area of Overlap refers to the area where the predicted bounding boxes (BBox) overlaps the label BBox, and Area of Union refers to the union of the predicted BBox and the label BBox. Based on IoU as the criteria, for each class, we can calculate the following parameters:
- *Confidence* Probability of each class prediction.
- *True positives (TP)* The prediction BBox with $\text {IoU}>0.5$ and meeting the category confidence threshold.
- *False positives (FP)* The prediction BBox with $\text {IoU}<0.5$ and meeting the category confidence threshold.
- *False negatives (FP)* ${\text {IoU}}=0$.

According to the above parameters, we have

2 $$\begin{aligned} \text {Precision}= & {} \dfrac{\text {TP}}{\text {TP+FP}}\end{aligned}$$

3 $$\begin{aligned} \text {Recall}= & {} \dfrac{\text {TP}}{\text {TP+FN}}. \end{aligned}$$

By setting different category confidence thresholds, we can obtain the Precision–Recall (PR) curve. Average precision (AP) is the area under the PR curve, and mAP is the average of all categories of AP. We have

4 $$\begin{aligned} \text {mAP} = \dfrac{\sum _{c=1}^{N}\text {AP}}{N}, \end{aligned}$$

where *N* is the total number of categories of class.

**Table 2:** Comparison of the experimental results with enhancement using SBE (proposed), Attention U-Net and R2U-Net.

Dataset	Enhancement method	B-Precision	B-Recall	M-Precision	M-Recall	mAP
BUS	SBE (proposed)	0.710	**0.846**	**0.865**	**1.000**	**0.788**
	Attention U-Net	**0.779**	0.846	0.644	1.000	0.712
	R2U-Net	0.666	0.845	0.756	1.000	0.711
BUSI	SBE (proposed)	**0.816**	**0.932**	0.789	**0.889**	0.802
	Attention U-Net	0.796	0.909	**0.814**	0.833	**0.805**
	R2U-Net	0.762	0.886	0.803	0.889	0.783
BUS+BUSI	SBE (proposed)	0.917	0.980	**0.888**	**0.963**	**0.902**
	Attention U-Net	**0.951**	**1.000**	0.805	0.926	0.878
	R2U-Net	0.934	0.980	0.729	0.963	0.832

Significant values are in bold.

### Results

#### Comparison of the experimental results with different image enhancement methods

**Figure 3:**
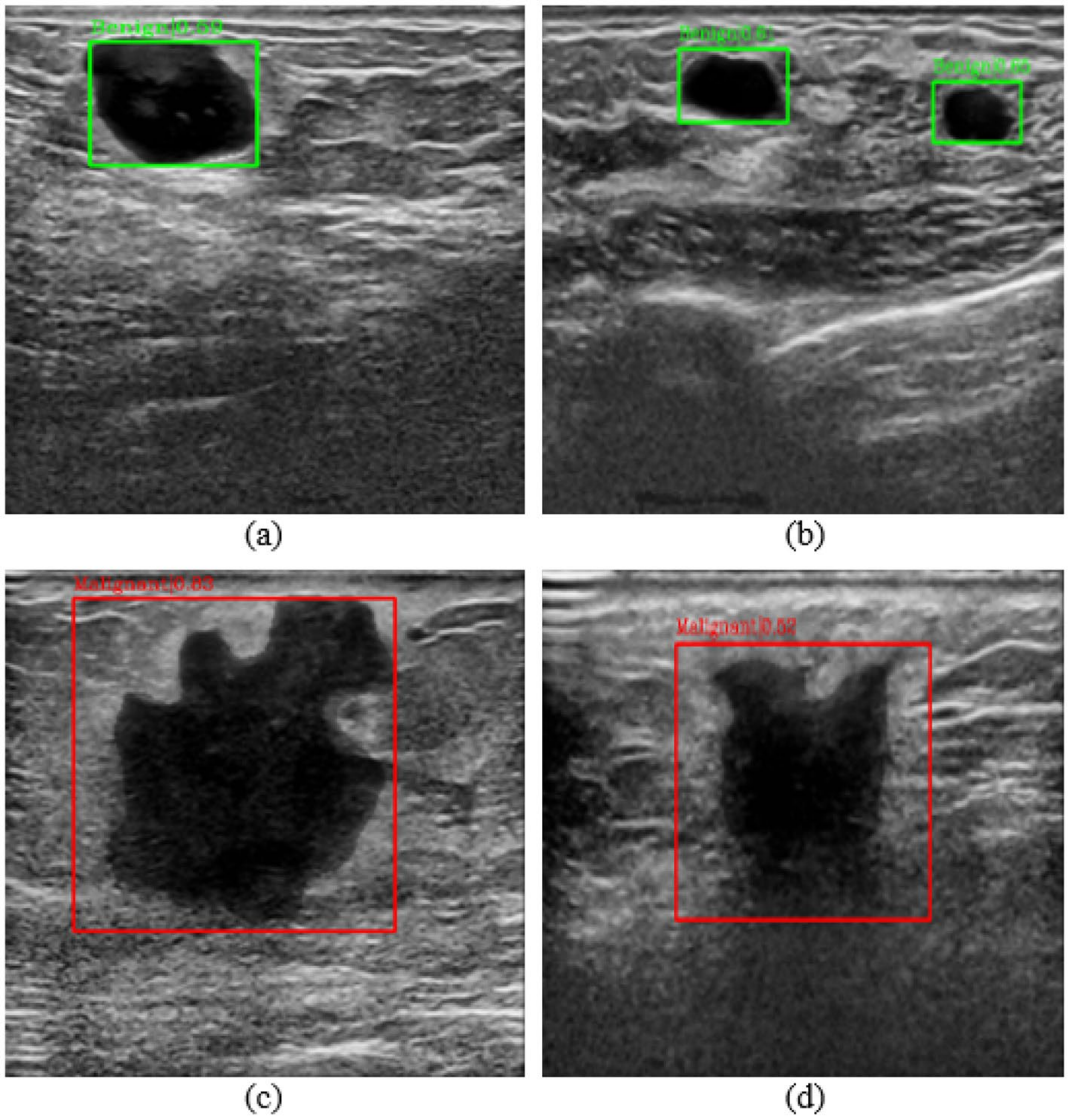
(**a,b**) Represents the results of benign lesions detected, including multiple lesions; (**c,d**) represents the results of malignant lesions detected.

**Figure 4:**
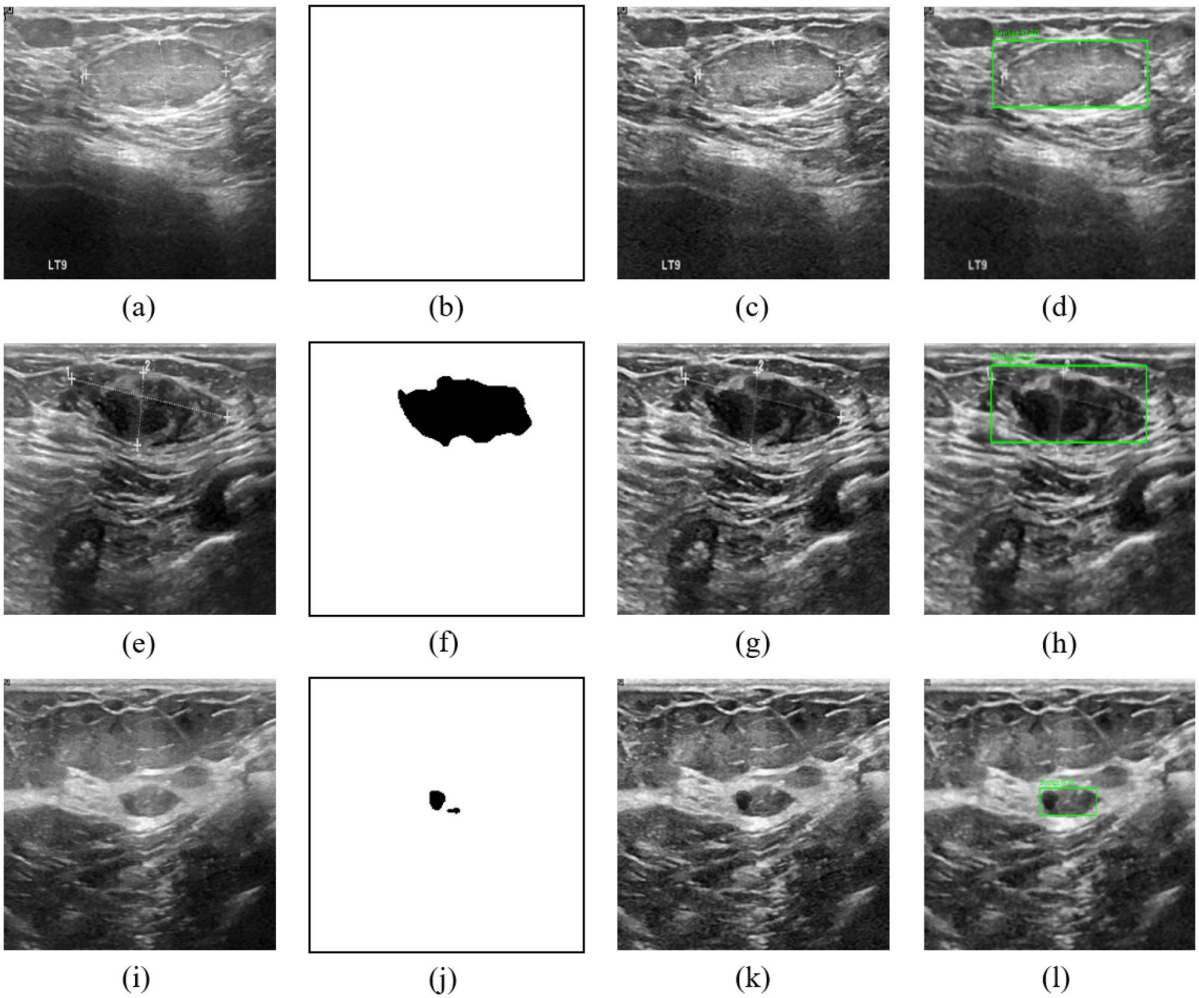
(**a,e,i**) Are the original images. (**b**) The lesion area was not segmented, (**f,j**) the lesion area was segmented incorrectly. (**c,g,k**) Are the results after SBE. (**d,h,l**) Are the detection results of FCOS.

**Figure 5:**
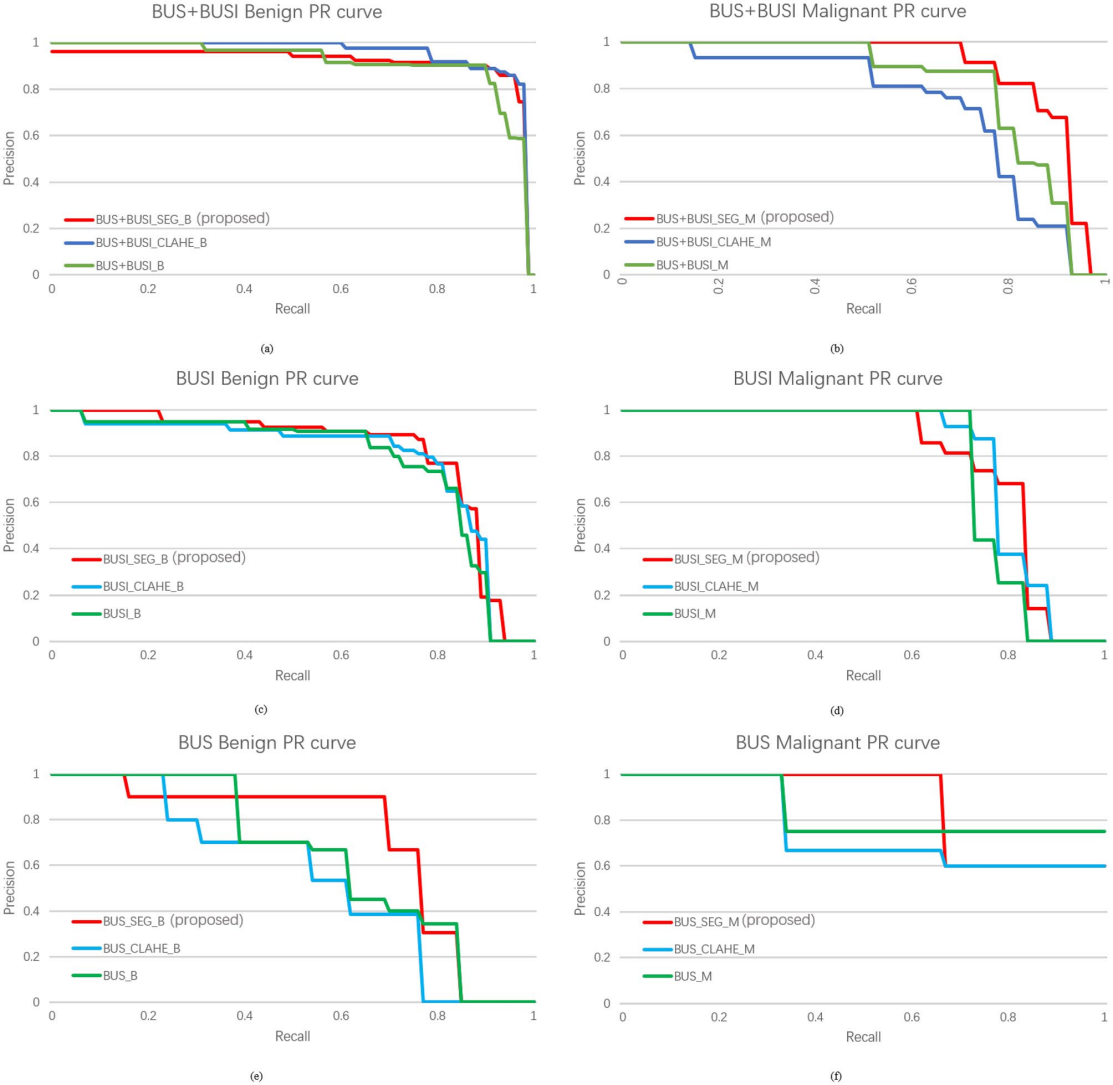
(**a,b**) BUS+BUSI datasets PR curve; (**c,d**) BUSI datasets PR curve; (**e,f**) BUS datasets PR curve.

We used different enhancement methods (our proposed method SBE, recurrent residual convolutional neural network based on U-Net (R2U-Net)^25^, Attention U-Net^26^, and traditional method contrast limited adaptive histogram equalization (CLAHE)^27^) and tested them based on both single dataset and composite dataset (BUS+BUSI). The experimental results are shown in Tables 2 and 3 and the PR curves are shown in Fig. 5. The results show that we have achieved 8 best mAP in 9 sets of comparative experiments. In malignant lesion detection preformance (M-Recall), we achieved all best results. Notice that the boundary of malignant tumors is usually irregular and the contrast between malignant tumors and normal tissue is low, so that the malignant tumors are not easy to detect. However, with our proposed SBE, the contrast is greatly enhanced, making malignant tumors easier to be detected. The experimental result images are shown in Fig. 3. We also found that during SBE, some breast lesions were not segmented (Fig. 4b), and some incorrect segmentations occurred (Fig. 4f,j). However, our method can still correctly detect the lesion areas, as shown in Fig. 4, which demonstrates good detection performance. Finally, for easy viewing, we surround the predicted benign tumors with a green box and the predicted malignant tumors with a red box.

**Table 3:** Comparison of breast cancer screening results using different enhancement methods.

Dataset	Enhancement method	B-Precision	B-Recall	M-Precision	M-Recall	mAP
BUS	SBE (proposed)	**0.710**	**0.846**	**0.865**	**1.000**	**0.788**
	CLAHE	0.552	0.769	0.756	1.000	0.654
	None	0.633	0.846	0.834	1.000	0.734
BUSI	SBE (proposed)	**0.816**	**0.932**	0.789	**0.889**	**0.802**
	CLAHE	0.787	0.909	**0.796**	0.889	0.792
	None	0.778	0.909	0.76	0.833	0.769
BUS+BUSI	SBE (proposed)	0.917	**0.980**	**0.888**	**0.963**	**0.902**
	CLAHE	**0.953**	0.980	0.727	0.926	0.840
	None	0.914	0.980	0.812	0.926	0.863

Significant values are in bold.

#### Comparison of the experimental results with different detection networks

To further verify the performance of our proposed method (i.e., combining FCOS with SBE), we compared it with a breast cancer ultrasound detection method proposed by Mo et al.^28^ in 2020. This method used YOLO V3 as the detection network and maked two changes to the original YOLO V3. First, Ref.^28^ adopted the K-Means++ algorithm and K-Mediods algorithm to optimize the original K-Means algorithm to set the anchor size. Second, the residual structure in the original YOLO V3 was changed, and a new residual network based on ResNet and DenseNet^29^ was constructed. We implement the method proposed by Ref.^28^ using our dataset for experimentation. We have obtained three different anchor size through K-Means++ and K-Mediods, and named the network that changed the anchor size as YOLO V3-anchor. Three sets of anchors The sizes are (34, 45), (40, 45), (40, 54), (60, 80), (66, 109), (88, 99), (90, 99), (94, 217), (164, 220) for BUS+BUSI; (25,50), (35, 69), (76, 62), (89, 128), (95, 100), (107, 192), (164, 220), (187, 341), (196, 208) for BUSI; (26, 27), (29, 59), (31, 78), (40, 54), (48, 57), (60, 80), (62, 134), (162, 134), (201, 361) for BUS. We reproduced a new residual structure according to the method proposed by Ref.^28^ and named it as YOLO V3-res. The experimental results are shown in Table 4. Notice that the performance of our method is not the best in all cases. However, as shown in Table 4, our method achieves the best results on both Precision and Recall of the detection of malignant lesions. More importantly, our method achieves the best results on the mAP performance measure.

**Table 4:** Comparison of the results of breast cancer detection experiments between our method and Ref.^28^.

Dataset	Method	B-Precision	B-Recall	M-Precision	M-Recall	mAP
BUS	Proposed	0.710	0.846	**0.865**	**1.000**	**0.788**
	YOLO V3-anchor	**0.897**	**0.923**	0.554	0.667	0.726
	YOLO V3-res	0.746	0.769	0.723	1.000	0.735
BUSI	Proposed	0.816	0.932	**0.789**	**0.889**	**0.802**
	YOLO V3-anchor	**0.898**	**0.954**	0.639	0.833	0.769
	YOLO V3-res	0.851	0.886	0.637	0.889	0.745
BUS+BUSI	Proposed	0.917	**0.980**	**0.888**	**0.963**	**0.902**
	YOLO V3-anchor	**0.938**	0.980	0.605	0.851	0.772
	YOLO V3-res	0.921	0.941	0.577	0.703	0.749

Significant values are in bold.

## Discussion

The above results show that our breast lesion detection system can detect the lesion region and classify the benign and malignant regions. When building this system, we mainly research two aspects. The first is the preprocessing of breast ultrasound images. We compared the effects of images under different enhancement methods on the detection results, including no enhancement, CLAHE, and SBE. After comparison, we found that the image processed by SBE can better improve the detection performance. Moreover, it can be proved that good local enhancement is helpful to the detection system. At the same time, we designed a new segmentation network. This network combines the characteristics of R2U-Net and Attention U-Net, and integrates the recurrent mechanism and attention mechanism into the network. The results show that the images enhanced by our network have achieved the best detection results on a variety of datasets. Second, we research the application of anchor-free detection network in breast lesion detection. We use YOLO V3 as a comparison network to prove the effectiveness of the anchor-free detection network in breast detection. In a variety of datasets, anchor-free detection network can achieve the highest mAP.

## Conclusions

This paper proposes an automatic breast cancer ultrasound image detection method based on deep learning, using anchor-free network FCOS as a breast cancer detection network, which can determine the location of breast cancer lesions and identify benign versus malignant. Our method can assist doctors in diagnosing breast lesions during ultrasound breast cancer screening, automatically locating lesions and classifying them (i.e., benign or malignant). We also propose a segmentation-based ultrasound image enhancement method to improve the breast cancer detection method’s performance. We use three public datasets, which are obtained from 8 different ultrasound acquisition devices, to compare our proposed method with anchor-basde method. Our proposed method can reach an mAP of 0.902, which demonstrates that our proposed method has good generalization ability and high clinical application value.

## Methods

This section covers image preprocessing methods of breast ultrasound images, an anchor-free detection network, and implementation process of our experiment.

In this study, we used data from three publicly available datasets, and our study is carried out in accordance with relevant guidelines and regulations.

### Image preprocessing

Due to the low contrast of ultrasound images and a large amount of speckle noise, appropriate preprocessing methods are essencial for subsequent image analysis. In this study, the preprocessing of ultrasound images consists of three steps. The first is to use traditional methods to enhance the contrast of the image and then denoise. Finally, we use our SBE method to further enhance the image’s contrast.

#### Traditional methods

We use CLAHE to enhance the image. The algorithm of CLAHE is as follows.

*Step I* First, divide the original picture into $\text {N}\times \text {N}$ subregions, and calculate the cumulative distribution function $\text {CDF}_{i}$, histogram $\text {Hist}_{i}$, and mapping function $\text {n}_{i}$ of the histogram in each subregion. We have,

5 $$\begin{aligned} \text {Hist}_{i}= & {} \dfrac{{d}(\text {CDF}_{i})}{{d}i}\end{aligned}$$

6 $$\begin{aligned} \text {n}_{i}= & {} \dfrac{255\times \text {CDF}_{i}}{\text {N}\times \text {N}}. \end{aligned}$$

Take the derivative of $\text {n}_{i}$ to get the slope *K* of the subregion. Set a threshold *T*, cut off the part of $\text {Hist}_{i}$ where *K* is greater than *T*, and evenly distribute it to the original image histogram to obtain a new histogram. Simultaneously, to avoid the blocking effect caused by the block operation, the bilinear interpolation method needs to be used to reconstruct each pixel’s gray value.

*Step II* The original image’s noise is enhanced for the ultrasound image calculated by CLAHE and the image needs to be denoised. Anisotropic diffusion^30^ is a denoising method based on partial differential equations, which can preserve image details while denoising.

Let $I_{p}^{t}$ denote the discrete sampling of the current image, *p* the coordinate of the sampled pixel, $I_{q}^{t}$ the neighborhood discrete sampling of $I_{p}^{t}$, $\partial _{p}$ denotes the neighborhood space of *p*, $|\partial p|$ denotes the size of the neighborhood space, and $\lambda$ control the diffusion strength. The iterative expression of anisotropic diffusion is

7 $$\begin{aligned} I_{p}^{t+1}=I_{p}^{t}+\frac{\lambda }{|\partial p|} \sum _{q \in \partial _{p}} c\left( I_{p}^{t}-I_{q}^{t}\right) \cdot \left( I_{p}^{t}-I_{q}^{t}\right) . \end{aligned}$$

Let *k* be the gradient threshold, then $c(I_{p}^{t}-I_{q}^{t})$ is

8 $$\begin{aligned} c(I_{p}^{t}-I_{q}^{t})=e^{-\left( \frac{(I_{p}^{t}-I_{q}^{t})}{k}\right) ^{2}}. \end{aligned}$$

Anisotropic diffusion needs to set the number of iterations *n*, gradient threshold *k*, and diffusion strength $\lambda$ to adjust the denoising effect.

#### Segmentation-based enhancement method

**Figure 6:**
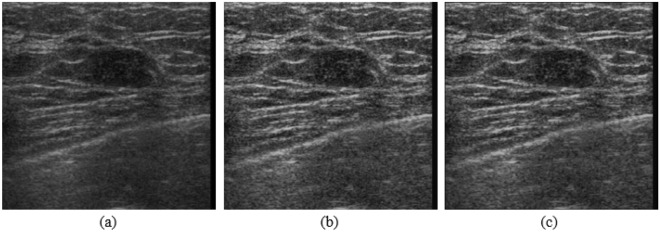
(**a**) Original ultrasound image; (**b**) image after CLAHE; (**c**) image after anisotropic diffusion.

After CLAHE and anisotropic diffusion, we obtain the contrast-enhanced image, as shown in Fig. 6. However, we found that the contrast of ultrasound images was still low. Therefore, we develop a segmentation-based enhancement method to further enhance the contrast of ultrasound images.

**Figure 7:**
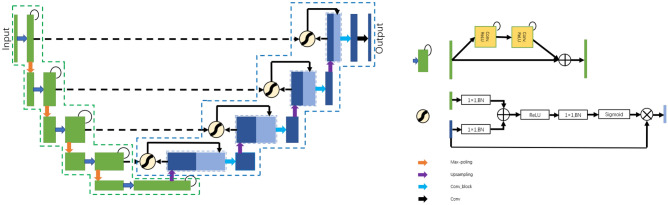
Structure of R2AttU-Net for segmentation. The structure of the dotted green line is from R2U-Net^25^. The structure of the dotted blue line is from Attention U-Net^26^.

**Figure 8:**
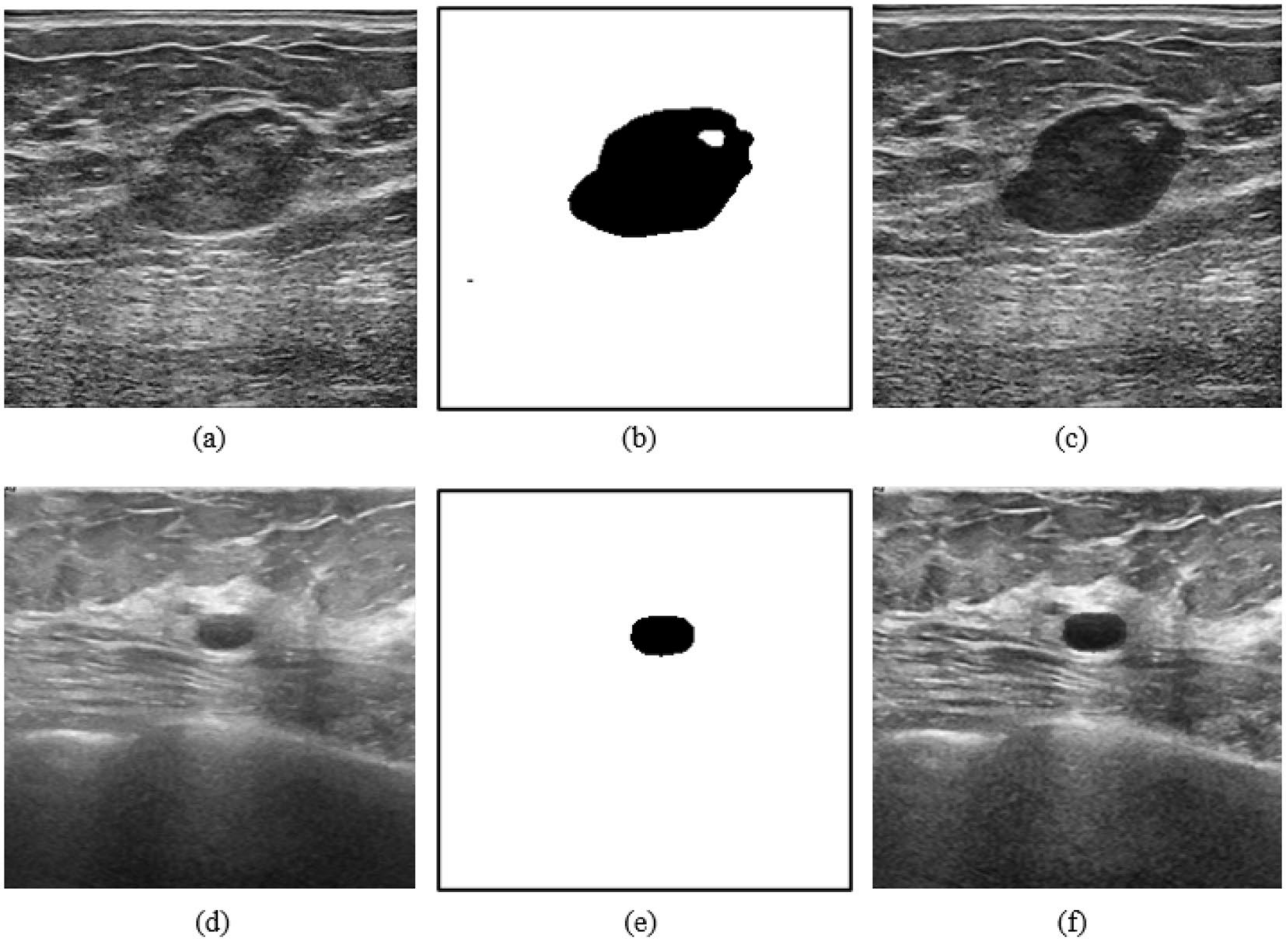
(**a,d**) Original ultrasound image; (**b,e**) output image of R2AttU-net; (**c,f**) enhanced image.

We integrated R2U-Net and Attention U-Net and designed R2AttU-Net. The downsampling part of R2AttU-Net is from R2U-Net, and the upsampling part is from Attention U-Net. R2AttU-Net network structure is shown in Fig. 7. We use BUSIS as training data of R2AttU-net and BUS and BUSI as test datas. We input the original ultrasound image (as shown in Fig. 8a) into R2AttU-net. After processing by R2AttU-net, the image in Fig. 8b is generated. Set the white part in Fig. 8b to 1 and the black part to 0.6, and multiply the image in Fig. 8b with the image in Fig. 8a to obtain a contrast-enhanced image shown in Fig. 8c. From Fig. 8, it can be seen that the contrast of the ultrasound image is substantialy enhanced.

**Figure 9:**
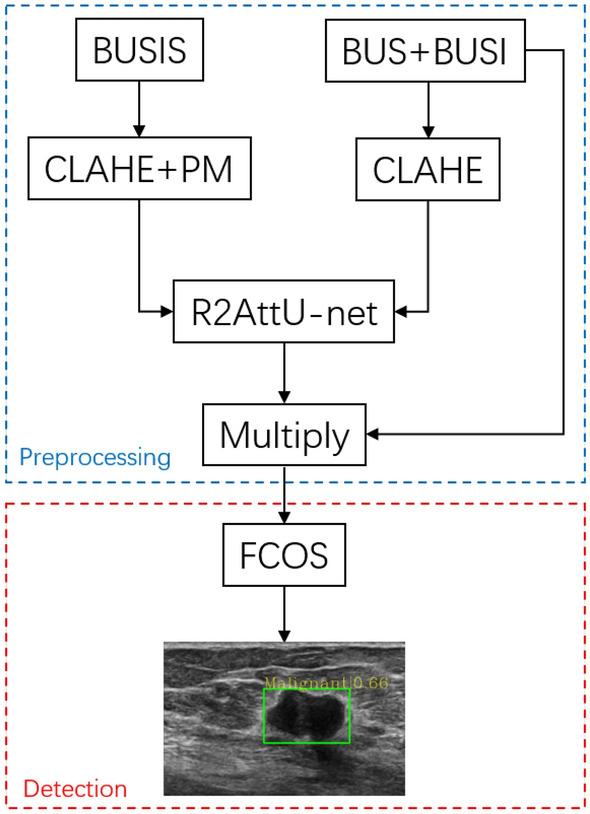
Flowchart of the proposed breast cancer detection method. The blue dotted line is preprocessing stage, and the red dotted line is detection stage.

**Figure 10:**
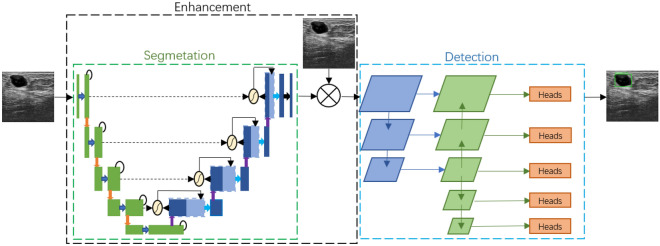
Breast cancer ultrasound detection network structure. Green box for segmentation network, black box for enhancement process, and blue box for detection network.

### Implementation

#### Lesion detection

Through the steps described above, we have obtained the enhanced image. In this section, we will introduce the last step of the whole breast lesion detection process.

#### Detection network

We adopted an anchor-free detection network, FCOS, as the detection network for breast lesions. FCOS outputs five sizes of heads to facilitate object detection of different sizes. Three loss functions (classification loss, center-ness loss and regression loss) are used to calculate the loss of the object category, center point, and bounding-box size, respectively. Compared with anchor-based object detection networks (such as Faster R-CNN, YOLO V3), anchor-free networks do not need to set anchor boxes in advance, so that can significantly reduce the number of parameters and reduce the large number of calculations due to anchor boxes (For example, the intersection over union (IoU) calculation and matching of anchor boxes and ground-truth boxes in training). These advantages over anchor-based object detection networks lead to faster detection and simpler training process in FCOS.

**Table 5:** Hyperparameters of R2AttU-Net and FCOS.

	R2AttU-Net	FCOS
Learning rate	0.001	0.001
Optimizer	Adam	Adam
Batch size	4	4
Epoch	200	300

The overall experimental steps of this study is shown in Fig. 9. In Fig. 10, we show our experimental steps in the form of a network structure. BUSI dataset includes 697 images containing lesions, but we found some duplicate images. We deleted the duplicate images and selected 610 breast ultrasound images from BUSI. Finally, we obtained a total of 773 images from the BUS dataset and the BUSI dataset. All breast ultrasound images were randomly selected for training data, validation data, and testing data according to the ratio of 8:1:1 and resized to $224 \times 224$.

We used FCOS based on the mmdetection object detection toolbox^31^. Using ResNet50^32^ as a backbone of FCOS, a total of 300 epochs are trained. The FCOS output detection box coordinates are mapped to the original breast ultrasound image and the final output result is obtained. We feedback/map the detection boxes to the original image, rather than the enhanced image, to avoid the segmentation results from interfering with the doctor’s diagnosis. The hyperparameters of the R2AttU-Net used in the image preprocessing stage and the FCOS used in the breast lesion detection stage are shown in Table 5.

## Data Availability

The datasets analysed during the current study are available in https://scholar.cu.edu.eg/?q=afahmy/pages/dataset, https://ieeexplore.ieee.org/abstract/document/goo.gl/SJmoti, and http://cvprip.cs.usu.edu/busbench.

## Abbreviations

AP: Average precision
BUS: Breast ultrasound
BUSI: Breast ultrasound image dataset
BUSIS: Breast ultrasound image segmentation dataset
CLAHE: Contrast limited adaptive histogram equalization
DL: Deep learning
FCOS: Fully convolutional one-stage object detection
mAP: Mean average precision
PR: Precision–Recall
R2U-Net: Recurrent residual convolutional neural network based on U-Net
SBE: Segmentation-based enhancement
SSD: Single shot multibox detector
YOLO: You only look once
